# Editorial: Application of epigenomics data to improve human and livestock health

**DOI:** 10.3389/fgene.2023.1221035

**Published:** 2023-05-26

**Authors:** Xiao Wang, Ying Yu, Lingzhao Fang, Zhimin Wang

**Affiliations:** ^1^ Institute of Animal Science and Veterinary Medicine, Shandong Academy of Agricultural Sciences, Jinan, China; ^2^ Konge Larsen ApS, Kongens Lyngby, Denmark; ^3^Laboratory of Animal Genetics and Breeding, Ministry of Agriculture and Rural Affairs of China, National Engineering Laboratory of Animal Breeding, College of Animal Science and Technology, China Agricultural University, Beijing, China; ^4^ Center for Quantitative Genetics and Genomics, Aarhus University, Aarhus, Denmark; ^5^Division of Endocrinology and Metabolic Diseases, The First Affiliated Hospital of Zhengzhou University, Zhengzhou, China

**Keywords:** epigenetics, epigenomics data, health improvement, human, livestock

With rapid advancements in next-generation sequencing technology, an enormous amount of epigenomic sequencing data is generated and helps us identify the epigenomic biomarkers and interpret biological mechanisms underlying complex health traits in human and livestock.

In Chinese Yorkshire pigs, Wang et al. reported associations of meat quality traits with DNA methylation and identified several candidate genes associated with these traits, such as *NCAM1*, *MED13*, and *TRIM37*. Rodriguez-Casanova et al. identified the promoter hypermethylation of *WNT1* in cfDNA as a potential noninvasive biomarker for luminal B breast cancer that supported the application of Infinium MethylationEpic array to identify new epigenetic noninvasive biomarkers in breast cancer.

Based on the combined RRBS DNA methylome and transcriptome, Huang et al. performed a genome-wide comparison of DNA methylation and gene expression in *Clostridium perfringens* (Cp) type C-infected resistant and susceptible piglets. Such integrative analysis identified 168, 198, and 7 mRNAs, showing inverse correlations between methylation and expression with Cp infection, and revealed that the differentially expressed (DE) genes *LBP*, *TBX21*, and *LCN2* were likely involved in the piglets against Cp infection.

As microRNA (miRNA) plays a key role in gene regulation, Li et al. found that miR-208b expression increased in C2C12 cells but Mettl8 expression decreased significantly, while Myh4 expression decreased and Myh7 expression increased. Zou et al. identified the common targets and the transcript levels of miR-223-3p, miR-122-5p, and miR-93-5p in polycystic ovarian syndrome (PCOS) rat ovaries.

For circulating miRNAs, miR-1-3p participates in myocardial apoptosis, and its upregulation in circulation is a direct and powerful indicator of fetal ventricular septal defect (Yang Y. et al.). Ma et al. reported the expression of circular RNA (circRNA) circ_0059706 in *de novo* acute myeloid leukemia and its association with prognosis. In animals, circRNAs may interact with miRNAs to further regulate mRNA to regulate sperm motility in Yili geese, including 20 circRNAs, 18 miRNAs, and 177 mRNAs targeting ppy-mir-16, hsa-mir-221-3p, gga-mir-499-5p, etc (Wu et al.).

The long non-coding RNAs (lncRNAs) are engaged in vital biological regulatory processes. Jiang et al. established a prognostic risk model with 10 lncRNAs and obtained a good predictive accuracy for overall survival of breast cancer individuals in both training and validation cohorts. In cows, Yang J. et al. detected 287 DE genes and 70 DE lncRNAs, where lncRNAs adjacent to the somatic cells count and somatic cell score QTLs influenced the mastitis pathogenesis by upregulating the expression of *TLR4*, *NOD2*, *CXCL8*, and *OAS2* genes.

As a dynamic and reversible RNA modification, N6-methyladenine (m^6^A) is involved in a wide range of biological and pathological processes. Gu et al. identified three different m^6^A sub-types including 27 samples in sub-type C1, 21 samples in sub-type C2, and 58 samples in sub-type C3. Li et al. identified 1,565 upregulated and 542 downregulated m^6^A methylation peaks with significant changes.

Histone post-translational modification is an essential epigenetic process controlling a variety of biological activities. Xie et al. hypothesized that lactylation of histones or non-histone appeared to engage in various biochemical processes to influence the biological reactions of the organism, when lactate reaches a specific level under a certain circumstance.

In summary, our Research Topic gathers the findings of identified epigenetic biomarkers (methylated genes, miRNAs, circRNAs, lncRNAs, and m^6^A) and reveals biological mechanisms using epigenomics data that could be used for further relevant studies.
